# Association Between Depression and Hepatic Steatosis According to Obese Status: The Korean National Health and Nutrition Examination Survey 2010–2019

**DOI:** 10.3390/medicina61091711

**Published:** 2025-09-19

**Authors:** Young Sang Lyu, Youngmin Yoon, Jin Hwa Kim, Sang Yong Kim

**Affiliations:** 1 Department of Endocrinology and Metabolism, Chosun University Hospital, Chosun University School of Medicine, Gwangju 61453, Republic of Korea; lyu0923@naver.com (Y.S.L.); endocrine@chosun.ac.kr (J.H.K.); 2 Division of Nephrology, Department of Medicine, Chosun University Hospital, Chosun University School of Medicine, Gwangju 61453, Republic of Korea; korean8503@chosun.ac.kr

**Keywords:** hepatic steatosis, hepatic steatosis, obesity

## Abstract

*Background and Objectives*: Hepatic steatosis is associated with an increased risk of liver-related morbidity and mortality. Although numerous studies have reported associations between depression, obesity, metabolic syndrome, and cardiovascular disease, the relationship between depression and hepatic steatosis has not yet been fully elucidated. Moreover, obesity is a shared risk factor for hepatic steatosis and depression; however, few studies have adequately adjusted for obesity as a potential confounder. In this study, we investigated the association between depression and hepatic steatosis stratified by obese and non-obese status. *Materials and Methods*: This study used data from the Korean National Health and Nutrition Examination Survey, conducted by the Korean Ministry of Health and Welfare between 2010 and 2019, which was a cross-sectional and nationally representative study of non-institutionalized civilians using a stratified, multistage, clustered probability sampling design. Multivariate logistic regression analyses were conducted to evaluate the association between depression and hepatic steatosis in the groups stratified by obese status. *Results*: Of 80,861 participants, data from 45,307 were included in the analysis. The prevalence of non-obese and obese hepatic steatosis was 3.1% and 19.3%, respectively, and the prevalence of diagnosed depression was 4.6%. Individuals with hepatic steatosis showed less favorable metabolic profiles, including higher rates of diabetes and elevated liver enzyme levels. Those with depression were older, predominantly female, and had lower socioeconomic status. After fully adjusting for confounding factors, multivariate logistic regression analysis showed that non-obese hepatic steatosis was significantly associated with an increased risk of depression, and obese hepatic steatosis was significantly associated with suicidal ideation and attempts. *Conclusions*: This study suggests a significant association between depression and hepatic steatosis with and without obese status. Given the significant impact of hepatic steatosis on depression outcomes, healthcare providers should screen patients with hepatic steatosis for depression and provide appropriate treatment as needed.

## 1. Introduction

Hepatic steatosis is considered the most common chronic liver disease worldwide, affecting an estimated 25–30% of the global population [1]. Its prevalence continues to rise, driven by escalating rates of obesity, sedentary lifestyles, and unhealthy dietary patterns. If left untreated, hepatic steatosis can lead to metabolic dysfunction-associated steatohepatitis (MASH) and, in some cases, may progress to advanced fibrosis, cirrhosis, and hepatocellular carcinoma [2,3]. Beyond its hepatic manifestations, hepatic steatosis is strongly associated with systemic metabolic disorders such as insulin resistance, type 2 diabetes mellitus, dyslipidemia, and hypertension, and is linked to an increased risk of cardiovascular disease, liver-related complications, and death [4,5]. Therefore, early screening and timely interventions are essential.

The growing interest in the bidirectional relationship between hepatic and mental health has brought the interplay between hepatic steatosis and depression into sharp focus [6,7,8]. Depression is one of the most common psychiatric disorders worldwide and leads to functional impairment, reduced quality of life, and elevated suicide risk. South Korea has the highest suicide mortality rate among the Organisation for Economic Co-operation and Development (OECD) member countries, with 24.6 deaths per 100,000 people reported in 2020, more than double the OECD average of 11.0 per 100,000 [9]. Notably, the suicide rate is markedly higher among men; in 2022, the male suicide rate reached 35.3 per 100,000, which is more than twice the rate observed in women (15.1 per 100,000). Moreover, suicide risk increases with advancing age in both sexes [10]. These trends highlight the urgent need to deepen our understanding of the biological and psychosocial mechanisms underlying depression and elucidate how potential risk factors are linked to hepatic steatosis, thereby informing the development of tailored prevention and intervention strategies.

These two conditions share behavioral and sociodemographic risk factors, including physical inactivity, unhealthy diet, lower socioeconomic status, and social isolation [11,12]. Notably, obesity is a well-established and powerful risk factor for hepatic steatosis and depression. Consequently, any investigation into the independent association between these disorders must appropriately adjust for body mass index (BMI) or stratify analyses according to obesity status [13,14,15]. To date, few studies have systematically accounted for obesity when examining hepatic steatosis and depression, particularly for severe mental health outcomes, such as suicidal ideation and attempts. Large nationally representative Asian cohorts addressing this issue remain scarce.

The present study analyzed data from the Korean National Health and Nutrition Examination Survey (KNHANES) collected between January 2010 and December 2019 to determine whether hepatic steatosis is associated with depression, suicidal ideation, and suicide attempts after adjusting for obesity status. These findings aim to clarify the relationship between hepatic steatosis and mental health burden and to inform integrated strategies addressing both physical and psychological health.

## 2. Materials and Methods

### 2.1. Study Design and Data Source

This cross-sectional analysis was based on data from the KNHANES collected between 2010 and 2019. KNHANES is a nationally representative rolling survey of non-institutionalized civilians conducted annually by the Korea Disease Control and Prevention Agency [16]. A complex, stratified, multistage, clustered probability sampling design was used to ensure that the findings could be generalized to the South Korean population. Trained personnel obtained health interview data, performed physical examinations, and collected fasting blood samples according to standardized protocols.

We selected this period for the following reasons: (1) all variables required for our analysis, including hepatic steatosis index (HSI) components, physician-diagnosed depression, suicidal ideation, and suicide attempts, were consistently collected using the same standardized methods; (2) the period preceded the COVID-19 pandemic, thereby avoiding potential confounding from pandemic-related changes in mental health and lifestyle factors; and (3) inclusion of the maximum possible number of eligible participants allowed us to increase the statistical power of our analyses and enable potential subgroup analyses.

All procedures adhered to the Declaration of Helsinki, and this secondary analysis was approved by the Institutional Review Board of Chosun University Hospital (IRB No. 2024-12-008). The study was conducted and reported in accordance with the Strengthening the Reporting of Observational Studies in Epidemiology (STROBE) guidelines.

### 2.2. Study Population

**Figure 1:**
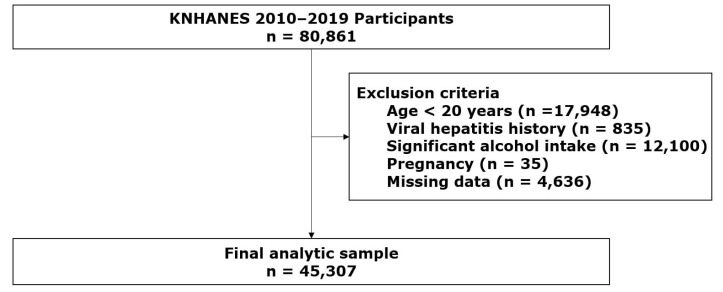
Flow chart of study participant selection.

Among 80,861 individuals surveyed during the study period, adults aged ≥20 years were eligible. Participants were excluded if they lacked key anthropometric, laboratory, or questionnaire data; reported viral hepatitis, significant alcohol intake (≥30 g/day in men or ≥20 g/day in women), or pregnancy; or provided implausible dietary energy intake (<500 or >5000 kcal/day). After exclusions, 45,307 participants were included in the final analytical sample (Figure 1).

### 2.3. Definition of Hepatic Steatosis and Obesity

Because liver imaging was not available in the KNHANES, steatosis was ascertained using the Hepatic Steatosis Index (HSI), calculated as follows:

Hepatic Steatosis Index (HSI) = 8 × (ALT/AST) + BMI (+ 2 if female) (+ 2 if diabetes).(1)

An HSI value > 36 was used to indicate hepatic steatosis [17]. Obesity status was determined using Asian BMI cut-offs, with non-obese defined as <25.0 kg/m^2^ and obese defined as ≥25.0 kg/m^2^. Accordingly, three mutually exclusive exposure groups were created: no hepatic steatosis, lean hepatic steatosis (HSI > 36 with BMI < 25.0 kg/m^2^), and obese hepatic steatosis (HSI > 36 with BMI ≥ 25.0 kg/m^2^).

### 2.4. Mental-Health Outcomes

The mental health variables were derived from self-reported items in the KNHANES Mental Health Module. Clinically diagnosed depression was recorded if the participants confirmed that a physician had ever diagnosed them with depression. Suicidal ideation was defined as answering “yes” to the question, “Have you seriously thought about committing suicide in the past year?”. A suicide attempt was determined when the participant answered “yes” to the question, “Have you actually attempted suicide in the past year?”

### 2.5. Covariates

A comprehensive set of covariates was selected a priori based on prior literature and biological plausibility. The demographic factors included age and sex. Socioeconomic variables included educational level (≤middle school vs. ≥high school), household income quartile, marital status, and residential area (urban vs. rural). Lifestyle factors encompassed smoking status (never, past, or current), alcohol consumption (≥one drink per month vs. less), regular physical activity (≥30 min of moderate activity at least twice per week), and total daily energy intake. The recorded comorbidities were hypertension, diabetes mellitus, hyperlipidemia, cardiovascular disease (stroke, myocardial infarction, or angina), asthma, chronic obstructive pulmonary disease, thyroid disease, malignancy, and renal insufficiency, each ascertained by self-reported physician diagnoses.

### 2.6. Statistical Analysis

All statistical procedures incorporated survey weights, strata, and clusters to account for the complex design of the KNHANES, thereby producing nationally representative estimates. Continuous variables are presented as weighted means ± standard errors and were compared with generalized linear modelling. Categorical variables are expressed as weighted percentages and were compared with Rao–Scott chi-square tests. Logistic regression models were fitted separately for depression, suicidal ideation, and suicide attempts with no hepatic steatosis as the reference category. Three progressive adjustment models were constructed: the first adjusted for age and sex; the second adjusted for socioeconomic and lifestyle factors; and the third incorporated comorbid conditions. The effect modification by obesity status was examined by including an interaction term between hepatic steatosis and obesity. Sensitivity analyses comprised repeating the main models after excluding participants with cardiovascular disease and separately redefining obesity based on waist circumference (≥90 cm in men or ≥85 cm in women). Adjusted odds ratios (ORs) with 95% confidence intervals (CIs) were calculated, and two-sided *p*-values < 0.05 were considered statistically significant. All analyses were performed using SPSS version 25.0 (IBM Corp., Armonk, NY, USA).

## 3. Results

### 3.1. Baseline Characteristics by Hepatic Steatosis Category

The clinical characteristics of the obese and non-obese subjects with hepatic steatosis are described in Table 1.

The study population was predominantly composed of individuals without hepatic steatosis, whereas lean hepatic steatosis constituted only a small fraction, and obese hepatic steatosis accounted for approximately one-fifth of the participants. A clear metabolic gradient was evident, with body mass index, waist circumference, blood pressure, fasting glucose, glycated hemoglobin, and liver enzyme concentrations increasing progressively from the non-hepatic steatosis group to the lean-hepatic steatosis group and were the highest in the obese-hepatic steatosis group. Patterns of cardiometabolic comorbidities mirrored these trends, with hypertension, diabetes, and dyslipidemia being the least common in non-hepatic steatosis and most common in obese hepatic steatosis. Lifestyle behaviors also varied, with regular physical activity being lowest among participants with lean hepatic steatosis, whereas current smoking and heavier alcohol use were more frequent in the hepatic steatosis group than in those without liver steatosis. Importantly, the prevalence of physician-diagnosed depression as well as reports of suicidal thoughts and suicide attempts increased in both hepatic steatosis phenotypes relative to the non-hepatic steatosis reference, suggesting an overlap between hepatic and mental health burdens even before full adjustment for confounders.

### 3.2. Characteristics by Depression Status

The characteristics of the study participants based on their depression status are described in Table 2.

Compared to their non-depressed counterparts, participants who reported a clinical diagnosis of depression were notably older and disproportionately female. They tended to live alone or in smaller households, occupied the lower end of the household income distribution, and had fewer years of formal education. Health behavior profiles also differed; depressed individuals were more likely to be physically inactive and to abstain from, or conversely, to engage more episodically in alcohol consumption. Overall caloric intake, together with absolute carbohydrate, protein, and fat consumption, was lower in the depressed group, consistent with the altered appetite or dietary patterns frequently observed in mood disorders. Comorbidities—including hypertension, diabetes, hyperlipidemia, and various respiratory or thyroid disorders—were more prevalent in the depressed cohort. Notably, both lean and obese hepatic steatosis appeared substantially more frequently in participants with depression than in those without, underscoring a potential convergence of metabolic liver disease and mental health vulnerabilities.

### 3.3. Association Between Hepatic Steatosis and Mental-Health Outcomes

The characteristics of the study participants based on their depression status are described in Table 3.

After adjusting for demographic, socioeconomic, lifestyle, and clinical factors, lean hepatic steatosis was independently associated with a higher likelihood of physician-diagnosed depression. In contrast, obese hepatic steatosis showed no clear association with depression but retained significant associations with suicidal ideation and suicide attempts. These patterns were consistent in the sensitivity analyses and did not differ significantly by sex or age.

## 4. Discussion

In this nationally representative sample of Korean adults, hepatic steatosis demonstrated distinct but clinically relevant associations with mental health outcomes after rigorous adjustments for demographic, socioeconomic, behavioral, and clinical covariates. Lean hepatic steatosis was independently linked to a higher likelihood of physician-diagnosed depression, whereas obese hepatic steatosis was not associated with depression diagnosis, but remained robustly related to suicidal ideation and suicide attempts. These patterns persisted across the sensitivity analyses and were consistent with sex and age, underscoring the psychiatric relevance of hepatic steatosis.

The present findings suggest that hepatic steatosis itself, rather than obesity alone, has mental health implications, although the specific manifestations (depressive disorder versus suicidality) may vary by obesity phenotype. Lean hepatic steatosis, despite lacking an overtly adverse anthropometric profile, harbors metabolic and hepatic risks that may be overlooked in routine practice [18]. Chronic subclinical inflammation and potential illness-related uncertainty in this group might predispose patients to depressive symptoms [19,20]. In contrast, obese hepatic steatosis, often accompanied by overt metabolic derangements and social stigma, appears to confer a heightened propensity toward suicidal thoughts and behaviors, perhaps via more profound psychosocial distress or neuroendocrine perturbations [21].

In our study, obesity-related hepatic steatosis was not associated with depression but was strongly related to suicidal ideation and suicide attempts. This may appear contradictory given that suicidal ideation and attempts are severe manifestations of depression. These association patterns may be explained by a combination of methodological and behavioral factors. First, differences in measurement approaches could be key contributors. Depression was assessed using self-reported physician diagnoses, whereas suicidal ideation and suicide attempts were evaluated using questionnaire-based self-reports, which may capture a broader and less clinically constrained spectrum of psychological distress. In addition, obese individuals may be less likely to disclose a depression diagnosis, potentially leading to underdiagnosis or underreporting [22,23]. Variations in healthcare-seeking behaviors among obese phenotypes could further influence diagnostic rates [21]. Finally, given the cross-sectional design, causality cannot be established. Longitudinal studies are needed to determine whether these differences reflect true mechanistic variations or diagnostic/reporting patterns.

Previous systematic reviews of observational studies have consistently reported positive associations between metabolic dysfunction-associated steatotic liver disease (MASLD) and depression [6,24,25]. For example, Gu et al. analyzed seven cross-sectional studies and found that MASLD was associated with a 13% higher risk of depression [6]. Similarly, Xiao et al. reviewed four cross-sectional studies and concluded that both the presence and severity of MASLD were positively associated with depression [25]. Feng et al. analyzed UK Biobank data and found that, while steatotic liver disease was initially linked to a higher risk of depression, this association disappeared after adjusting for BMI [26]. In line with these findings, Cho et al. reported that depression increased the risk of incident nonalcoholic fatty liver disease (NAFLD) in obese individuals [27] and Kim et al. demonstrated that depression was independently associated with NAFLD among adults in the United States [25,28]. Furthermore, Labenz et al. observed that NAFLD is associated with a higher risk of both anxiety and depression in a large population-based cohort [29]. However, these studies were limited by inadequate adjustment for key confounding factors such as socioeconomic status and a lack of evaluation of major depression-related outcomes, including suicidal ideation and suicide attempts. In contrast, our study strengthens the current evidence base by comprehensively accounting for major socioeconomic determinants that play a crucial role in depression risk, and further clarifies the relationship between hepatic steatosis and depression through stratified analyses according to obesity status.

Several mechanistic pathways can explain the association between hepatic steatosis and depression. First, hepatic steatosis induces chronic low-grade inflammation, characterized by elevated inflammatory cytokines (e.g., IL-6, TNF-α) and C-reactive protein (CRP), which can affect the central nervous system by altering neurotransmitter metabolism and impairing mood regulation [19,30]. Although these inflammatory markers are non-specific and may also be elevated in other conditions such as cardiovascular or neurological diseases, their consistent elevation in hepatic steatosis provides a unique insight into a possible shared biological pathway linking liver pathology and mental-health disturbances. In steatosis, chronic low-grade inflammation may originate from hepatic fat accumulation, lipotoxicity, and associated metabolic dysregulation, which can influence the central nervous system through neuroinflammatory processes, altered neurotransmitter metabolism, and hypothalamic–pituitary–adrenal axis activation. Highlighting this association underscores that the mental-health burden observed in hepatic steatosis may not be solely attributable to psychosocial or metabolic comorbidities, but could also be partly driven by liver-specific inflammatory processes—offering a potential biological target for integrated therapeutic strategies. Additionally, an imbalance in the gut-liver-brain axis may lead to changes in the gut microbiota, increased intestinal permeability, and translocation of endotoxins, triggering neuroinflammation and exacerbating depressive symptoms [31,32,33,34]. Insulin resistance, a common pathological feature, may further impair brain energy metabolism and neuronal function, contributing to the cognitive and emotional dysregulation associated with depression [4,20]. Notably, in obese patients with hepatic steatosis, the combination of metabolic syndrome [35], neuroendocrine alterations (e.g., hypothalamic–pituitary–adrenal axis activation [36]), and psychosocial stress [37,38] from social stigma may increase the risk of suicidal ideation and behaviors, whereas in lean hepatic steatosis, hidden disease burden and illness-related uncertainty may play a greater role in predisposing individuals to depressive symptoms [36]. These complex mechanisms underscore the need for deeper research and integrated management approaches at the intersection of liver and mental health.

Given that South Korea has the highest suicide mortality rates among the OECD nations, our finding of a significant association between obesity-related hepatic steatosis and suicidality has immediate public health relevance. Routine mental health screening, including direct questions about self-harm, should be considered in patients with hepatic steatosis, particularly in those with obesity. Conversely, in lean hepatic steatosis, clinicians should maintain a high index of suspicion for depressive symptoms even in the absence of obesity. Integrating liver and mental healthcare through collaborative care models or shared clinical pathways may enhance outcomes in this dual-burden population. At the policy level, public health strategies targeting lifestyle modification should incorporate mental health promotion and suicide prevention components, given their overlapping determinants.

Bariatric surgery, which is an effective intervention for sustained weight loss and metabolic improvement in individuals with obesity, has also been associated with a reduction in depressive symptoms. Meta-analyses and longitudinal studies have reported significant improvements in depression scores following bariatric procedures, with benefits observed as early as 6–12 months postoperatively that persist for several years [39,40]. These improvements are thought to result from multiple mechanisms, including substantial weight loss, amelioration of obesity-related comorbidities, enhanced self-esteem, and reduced weight-related stigma [41]. Given that obese hepatic steatosis was strongly associated with suicidality in our study, bariatric surgery could plausibly confer additional mental health benefits on this population by addressing both metabolic derangement and psychosocial stressors. However, careful patient selection and postoperative psychological follow-up remain essential because some studies have noted an increased suicide risk in certain subgroups after surgery [42].

The strengths of this study include the use of a large, nationally representative dataset; comprehensive adjustment for socioeconomic, behavioral, and clinical factors; and separate evaluation of depression, suicidal ideation, and suicide attempts. The analysis also employed contemporary hepatic steatosis nomenclature and Asian-specific BMI thresholds to enhance cultural and clinical relevance. This study has a few limitations that warrant consideration. First, its cross-sectional design precludes causal inferences. Therefore, the associations observed should be interpreted with caution. Future longitudinal or prospective studies are warranted to confirm these findings and clarify the temporal sequence and potential causal pathways underlying the observed relationships. Second, hepatic steatosis was evaluated using the FLI, which is based on surrogate biochemical and anthropometric markers, rather than direct imaging or histological confirmation. Although the FLI is a widely applied and validated tool for identifying hepatic steatosis in large-scale population research, it cannot differentiate between the different stages of liver disease, such as steatohepatitis or fibrosis. Third, depression and suicidality were self-reported and may have been subject to recall or reporting bias. Fourth, residual confounding by unmeasured variables, such as genetic risk scores, antidepressant use, or detailed diet quality, cannot be excluded. Fifth, the KNHANES questionnaire did not capture the depression severity or duration, which may have modified the observed associations. Sixth, both depression and suicidality were self-reported and may have been subject to recall or reporting bias. Seventh, in addition, we were unable to evaluate how the severity or stage of hepatic steatosis correlates with depression levels, as the KNHANES dataset did not include imaging- or histology-based assessments of liver disease. Thus, the potential influence of disease severity—including the psychological burden of diagnosis even at early stages—on depression could not be addressed in this study. Lastly, serum biomarkers linked to metabolic syndrome, hepatic steatosis, and mood disorders, such as butyrylcholinesterase (BChE), were not available in the KNHANES dataset and therefore could not be analyzed. Future research, including BChE measurements, may help clarify its relevance in the association between hepatic steatosis and depression [43,44].

## 5. Conclusions

In this cross-sectional study, hepatic steatosis was found to be associated with adverse mental-health outcomes, but the nature of these associations differed by obesity status: lean hepatic steatosis was more frequently associated with depression, whereas obese hepatic steatosis was more often associated with suicidal ideation and attempts. These findings underscore the need for integrated hepatometabolic and psychiatric screening and care regardless of body size. Concurrently addressing both liver and mental health may improve overall morbidity and help mitigate the substantial suicide burden in Korea. However, given the cross-sectional design and use of a population-specific non-invasive index to define hepatic steatosis, our findings cannot establish causality and may have limited generalizability to populations with different demographic or clinical characteristics. Further prospective studies with diverse populations are required to confirm these associations.

## Figures and Tables

**Table 1:** Clinical characteristics of hepatic steatosis subjects with obese and non-obese status.

	Non-Hepatic Steatosis	Lean Hepatic Steatosis	Obese Hepatic Steatosis	*p*-Value
*N* (%)	35,181 (77.6)	1425 (3.1)	8701 (19.3)	0.000
Sex (male, %)	43.4	57.6	53.7	0.000
Age (years)	46.38 ± 0.14	46.84 ± 0.45	46.93 ± 0.22	0.000
BMI (kg/m^2^)	22.47 ± 0.01	23.74 ± 0.03	28.70 ± 0.04	0.000
WC (cm)	78.10 ± 0.06	83.37 ± 0.17	93.64 ± 0.11	0.000
SBP (mmHg)	114.92 ± 0.45	117.68 ± 1.90	122.41 ± 0.61	
DBP (mmHg)	73.01 ± 0.30	74.79 ± 1.21	78.93 ± 0.41	
FPG (mg/dL)	95.01 ± 0.11	112.11 ± 1.11	107.69 ± 0.37	0.000
HbA1c (%)	5.59 ± 0.00	6.23 ± 0.04	6.05 ± 0.01	0.000
AST (IU/L)	20.68 ± 0.06	26.33 ± 0.42	26.05 ± 0.17	0.000
ALT (IU/L)	17.08 ± 0.06	43.59 ± 0.87	35.96 ± 0.39	0.000
Family income percentile (%) ^a^				0.000
<25	14.9	12.4	16.0	
25–50	24.6	25.2	25.8	
50–75	29.1	29.6	31.0	
≥75	31.4	32.8	27.3	
More than high school education (%)	39.8	39.5	36.0	0.000
Residence in urban area (%)	83.8	83.4	81.3	0.000
Marital statue				0.000
Never married	22.5	20.7	20.3	
Married-living together	67.6	69.1	68.2	
Married others	9.9	10.2	11.6	
Living status				0.209
Living alone	8.1	8.5	9.0	
Living with one family member	23.5	22.9	23.3	
Living with more than one member	68.4	68.6	67.7	
Occupation (Yes, %)	62.8	64.7	65.0	0.007
Smoking				0.000
Never	62.1	54.4	55.2	
Past	19.7	22.8	20.8	
Current	18.2	22.8	23.9	
Alcohol drinking (Yes, %)	55.3	51.4	52.0	0.000
Regular exercise ^a^ (yes, %)	31.9	25.4	30.4	0.000
Total energy (kcal)	1979.19 ± 7.21	2026.88 ± 27.65	2017.48 ± 14.02	0.000
Carbohydrate (g)	307.25 ± 1.04	316.85 ± 409	311.36 ± 1.92	0.000
Protein (g)	71.09 ± 0.33	74.84 ± 1.47	73.32 ± 0.63	0.000
Fat (g)	44.47 ± 0.28	45.69 ± 1.19	46.01 ± 0.57	0.000
Comorbidity (%)				
Hypertension	15.0	22.7	27.5	0.000
Diabetes mellitus	4.6	19.9	13.2	0.000
Hyperlipidemia	10.7	20.0	19.3	0.000
Cerebral infarction	1.5	1.6	2.0	0.000
Myocardial infarction	0.6	0.9	0.9	0.002
Angina	1.3	1.6	1.8	0.001
Pulmonary tuberculosis	4.1	2.5	2.5	0.000
Asthma	2.8	2.4	3.5	0.000
Renal insufficiency	0.3	0.4	0.3	0.013
COPD				0.000
Thyroid disease	3.4	4.0	3.6	0.007
Malignancy	3.2	2.7	2.4	0.000
Mental health				
Depression diagnosis	3.9	5.2	4.7	0.001
Suicidal idea	0.8	1.4	1.4	0.000
Suicidal attempt	0.4	0.7	0.5	0.000
HSI	30.27 ± 0.02	38.23 ± 0.07	40.64 ± 0.05	0.000

Continuous variables are presented as mean ± standard deviation, and categorical variables are reported as weighted percentages. BMI, body mass index; COPD chronic obstructive lung disease; DBP, diastolic blood pressure; FPG, fasting plasma glucose; HIS, Hepatic steatosis index; SBP, systolic blood pressure; WC, waist circumference. ^a^ Regular exercise was defined as participating in physical activity for at least 30 min on two or more occasions per week.

**Table 2:** Characteristics of study participants based on depression status.

	No Depression	Depression	*p*-Value
*N* (%)	43,252 (95.4)	2055 (4.6)	0.000
Sex (male, %)	46.8	23.6	0.000
Age (years)	46.31 ± 0.13	51.0 ± 0.43	0.000
BMI (kg/m^2^)	23.7 ± 0.02	24.00 ± 0.10	0.000
SBP (mmHg)	116.29 ± 0.40	118.20 ± 1.69	0.000
DBP (mmHg)	74.12 ± 0.26	74.46 ± 1.09	0.000
WC (cm)	81.33 ± 0.07	81.57 ± 0.30	0.000
FPG (mg/dL)	98.01 ± 0.13	99.44 ± 0.57	0.000
HbA1c (%)	5.70 ± 0.00	5.79 ± 0.02	0.000
AST (IU/L)	21.89 ± 0.06	22.66 ± 0.29	0.000
ALT (IU/L)	21.66 ± 0.11	21.67 ± 0.46	0.000
Family income percentile (%) ^a^			0.000
<25	14.5	28.1	
25–50	24.8	26.8		
0.00050–75	29.8	22.0	
≥75	30.9	23.2	
More than high school education (%)	39.8	23.0	0.000
Residence in urban area (%)	83.4	82.3	0.340
Marital statue			0.000
Never married	22.3	15.5	
Married-living together	67.9	64.0	
Married others	9.8	20.5	
Living status			0.000
Living alone	8.1	13.4	
Living with one family member	23.2	28.7	
Living with more than one member	68.7	57.9	
Occupation (Yes, %)	64.1	44.3	
Smoking			0.000
Never	60.2	67.4	
Past	20.2	15.1	
Current	19.5	17.3	
Alcohol drinking (Yes)	55.1	40.3	0.000
Regular exercise ^a^ (yes, %)	31.6	25.8	0.000
Total energy (kcal)	1998.86 ± 6.66	1743.70 ± 27.12	0.000
Carbohydrate (g)	309.51 ± 0.95	281.92 ± 3.79	0.000
Protein (g)	72.12 ± 0.31	60.79 ± 1.20	0.000
Fat (g)	45.16 ± 0.26	36.97 ± 0.97	0.000
Comorbidity (%)			
Hypertension	17.5	24.2	0.000
Diabetes mellitus	6.7	9.8	0.000
Hyperlipidemia	12.2	23.9	0.000
Cerebral infarction	1.5	3.3	0.000
Myocardial infarction	0.7	1.5	0.001
Angina	1.3	3.7	0.000
Pulmonary tuberculosis	3.7	4.9	0.041
Asthma	2.8	6.0	0.000
Renal insufficiency	0.3	0.8	0.000
COPD	0.4	0.7	0.000
Thyroid disease	3.4	6.2	0.000
Malignancy	2.9	4.5	0.002
Mental health			
Suicidal idea	0.7	6.4	0.000
Suicidal attempt	0.3	4.3	0.000
HSI	32.55 ± 0.03	33.18 ± 0.15	0.000
hepatic steatosis prevalence by HSI			0.001
Normal	77.2	73.1	
Lean hepatic steatosis	3.2	4.1	
Obese hepatic steatosis	19.6	22.9	

Continuous variables are presented as mean ± standard deviation, and categorical variables are reported as weighted percentages. BMI, body mass index; COPD chronic obstructive lung disease; FPG, fasting plasma glucose; HSI, Hepatic steatosis index; SBP, systolic blood pressure; WC, waist circumference. ^a^ Regular exercise was defined as participating in physical activity for at least 30 min on two or more occasions per week.

**Table 3:** Multivariate Odds Ratio for depression based on hepatic steatosis.

	Age, Sex-Adjusted Model		Multivariate Model 1		Multivariate Model 2	
	OR(95% Cl)	*p*-Value	OR(95% Cl)	*p*-Value	OR(95% Cl)	*p*-Value
Depression diagnosis						
Lean hepatic steatosis	1.400(1.083–1.810)	0.037	1.393(1.074–1.808)	0.000	1.463(1.078–1.985)	0.015
Obese hepatic steatosis	1.294(1.148–1.458)	0.000	1.176(1.038–1.332)	0.000	1.117(0.966–1.293)	0.136
Suicidal idea						
Lean hepatic steatosis	1.232(0.646–2.348)	0.526	1.400(0.730–2.686)	0.311	1.515(0.783–2.933)	0.217
Obese hepatic steatosis	1.394(1.086–1.788)	0.009	1.328(1.022–1.725)	0.034	1.320(1.006–1.730)	0.045
Suicidal attempt						
Lean hepatic steatosis	0.928(0.369–2.333)	0.874	1.071(0.427–2.690)	0.884	1.802(0.665–4.884)	0.487
Obese hepatic steatosis	1.076(0.798–1.452)	0.630	1.098(0.792–1.524)	0.575	1.519(1.016–2.272)	0.042

CI, confidence interval; OR, odds ratio. The multivariate model 1 was adjusted for age, sex, education level, marital status, economic status, smoking status, alcohol status, The multivariate model 2 was adjusted for Model 1 plus comorbidity.

## Data Availability

Information can be accessed from the Korea National Health and Nutrition Examination Survey (KNHANES), which is organized by the Korea Centers for Disease Control and Prevention (KCDCP). The data is freely available on the KCDCP website (https://knhanes.cdc.go.kr), accessed on 2 June 2025.

## Abbreviations

The following abbreviations are used in this manuscript:

KNHANES	Korean National Health and Nutrition Examination Survey
OECD	Organisation for Economic Co-operation and Development
BMI	body mass index
HSI	Hepatic steatosis index
STROBE	Strengthening the Reporting of Observational Studies in Epidemiology
OR	Odds ratio
CI	Confidence intervals
MASLD	Metabolic dysfunction-associated steatotic liver disease
NAFLD	Nonalcoholic fatty liver disease
CRP	C-reactive protein
BChE	butyrylcholinesterase
